# Supplementary material for: Redistribution of the SWI/SNF Complex Dictates Coordinated Transcriptional Control over Epithelial–Mesenchymal Transition of Normal Breast Cells through TGF-β Signaling
Source: Cells. 2022 Aug 24;11(17):2633. doi: 10.3390/cells11172633 (PMC9454592; doi:10.3390/cells11172633)
Supplement: Supplementary file 1 [file cells-11-02633-s001.zip › cells-1847090-supplementary.pptx]

## Slide 1
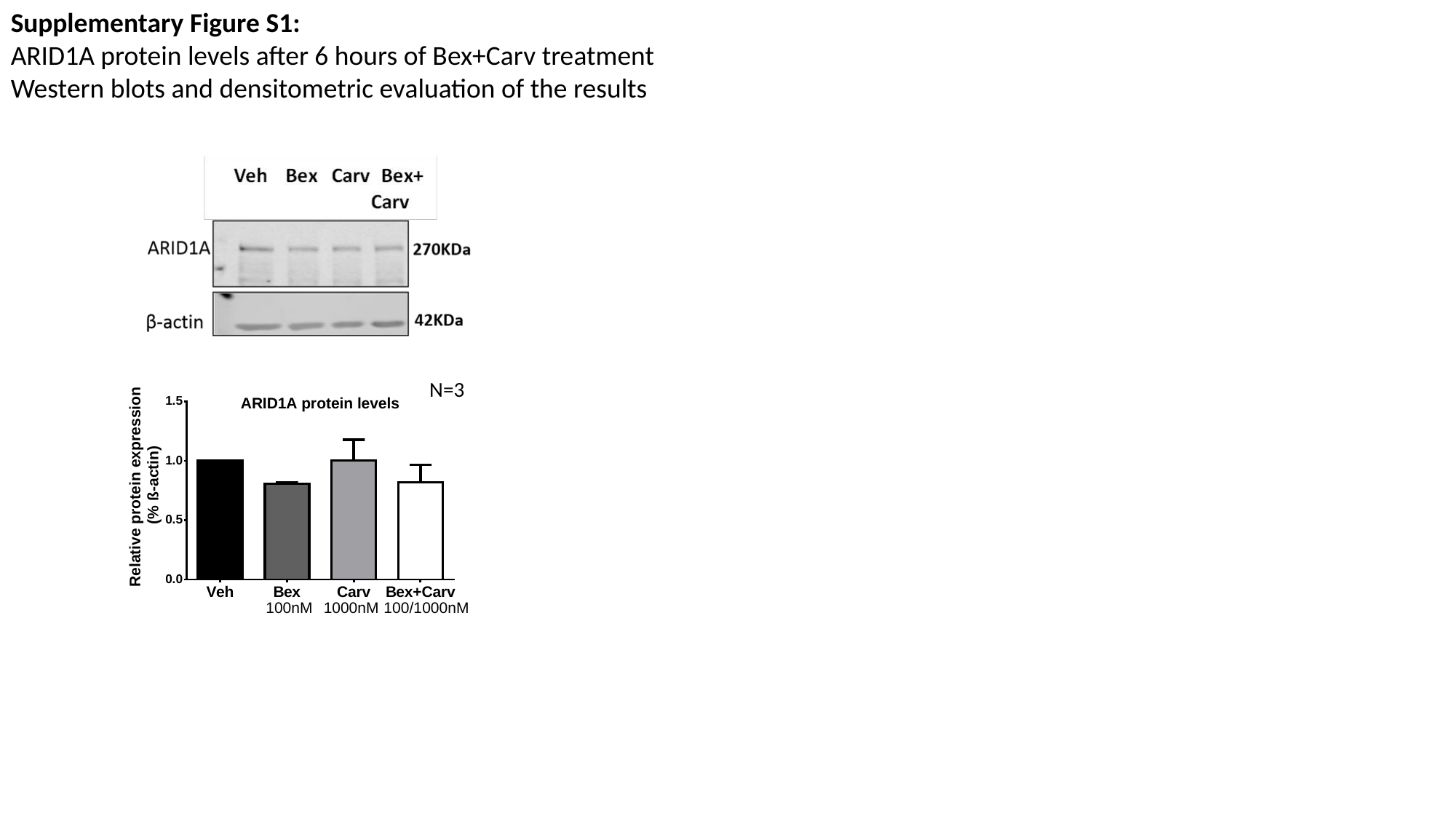

Supplementary Figure S1:
ARID1A protein levels after 6 hours of Bex+Carv treatment
Western blots and densitometric evaluation of the results
N=3

## Slide 2
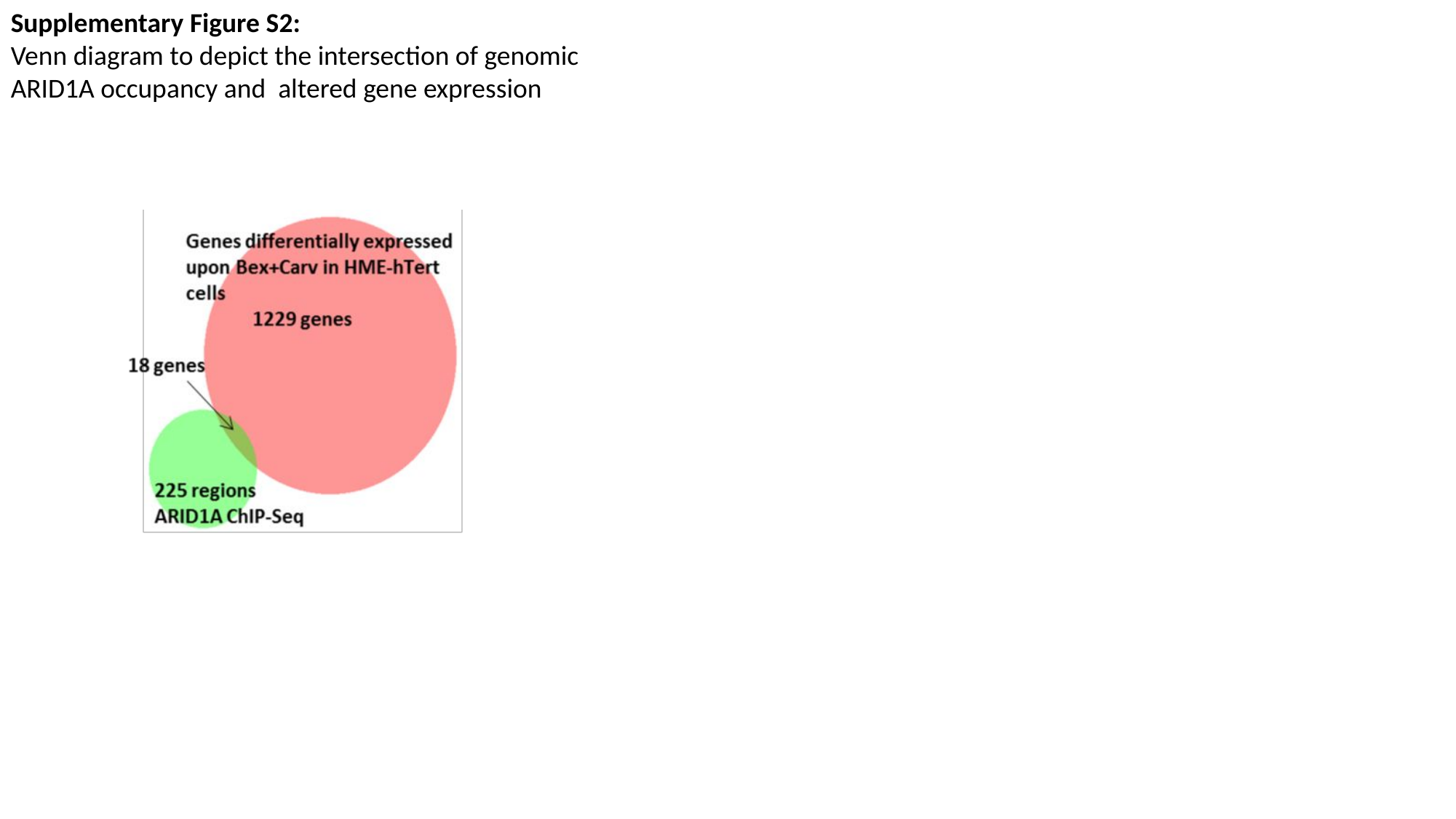

Supplementary Figure S2:
Venn diagram to depict the intersection of genomic
ARID1A occupancy and altered gene expression

## Slide 3
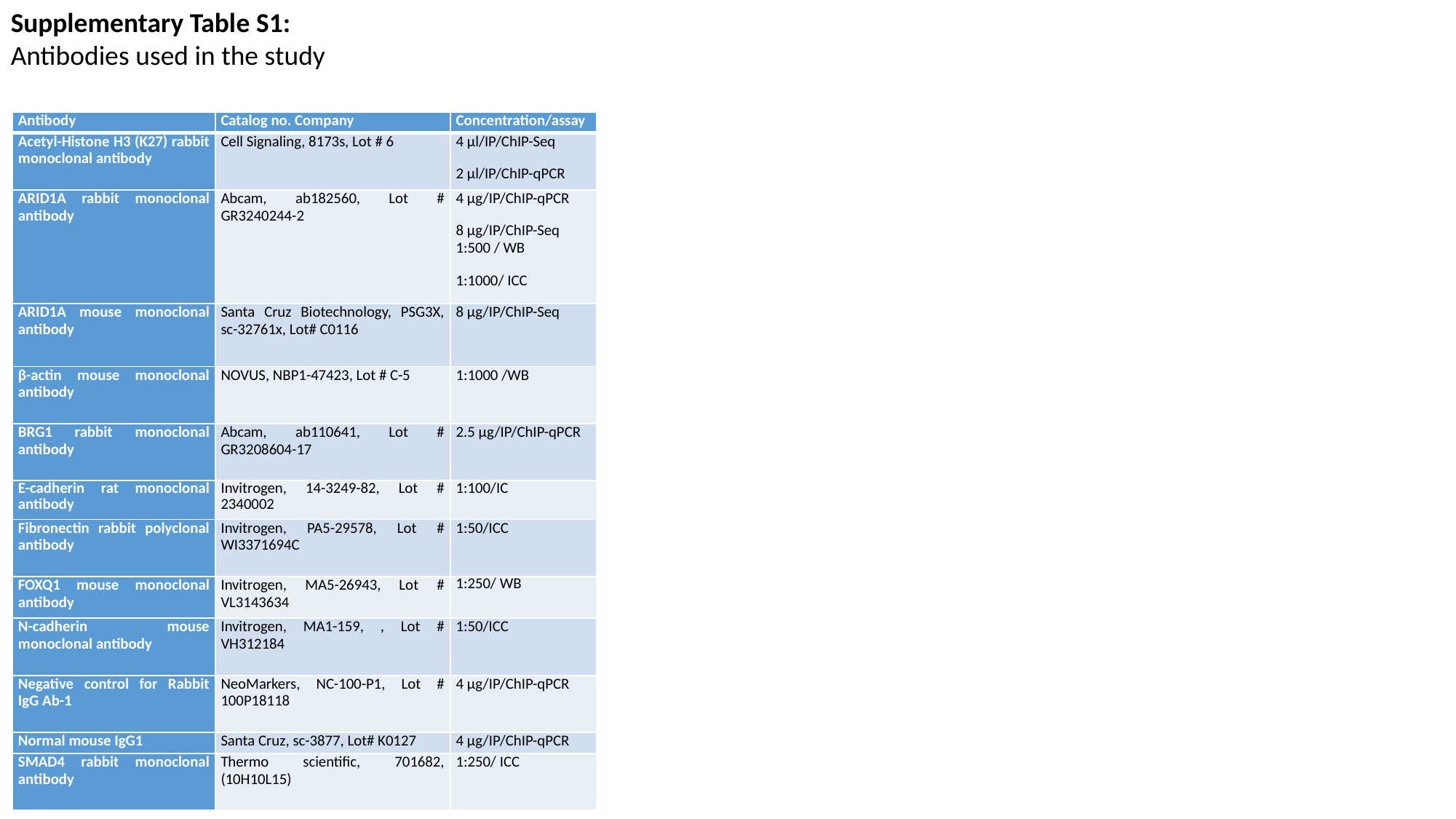

Supplementary Table S1:
Antibodies used in the study
| Antibody | Catalog no. Company | Concentration/assay |
| --- | --- | --- |
| Acetyl-Histone H3 (K27) rabbit monoclonal antibody | Cell Signaling, 8173s, Lot # 6 | 4 µl/IP/ChIP-Seq 2 µl/IP/ChIP-qPCR |
| ARID1A rabbit monoclonal antibody | Abcam, ab182560, Lot # GR3240244-2 | 4 µg/IP/ChIP-qPCR 8 μg/IP/ChIP-Seq 1:500 / WB 1:1000/ ICC |
| ARID1A mouse monoclonal antibody | Santa Cruz Biotechnology, PSG3X, sc-32761x, Lot# C0116 | 8 μg/IP/ChIP-Seq |
| β-actin mouse monoclonal antibody | NOVUS, NBP1-47423, Lot # C-5 | 1:1000 /WB |
| BRG1 rabbit monoclonal antibody | Abcam, ab110641, Lot # GR3208604-17 | 2.5 µg/IP/ChIP-qPCR |
| E-cadherin rat monoclonal antibody | Invitrogen, 14-3249-82, Lot # 2340002 | 1:100/IC |
| Fibronectin rabbit polyclonal antibody | Invitrogen, PA5-29578, Lot # WI3371694C | 1:50/ICC |
| FOXQ1 mouse monoclonal antibody | Invitrogen, MA5-26943, Lot # VL3143634 | 1:250/ WB |
| N-cadherin mouse monoclonal antibody | Invitrogen, MA1-159, , Lot # VH312184 | 1:50/ICC |
| Negative control for Rabbit IgG Ab-1 | NeoMarkers, NC-100-P1, Lot # 100P18118 | 4 µg/IP/ChIP-qPCR |
| Normal mouse IgG1 | Santa Cruz, sc-3877, Lot# K0127 | 4 μg/IP/ChIP-qPCR |
| SMAD4 rabbit monoclonal antibody | Thermo scientific, 701682, (10H10L15) | 1:250/ ICC |

## Slide 4
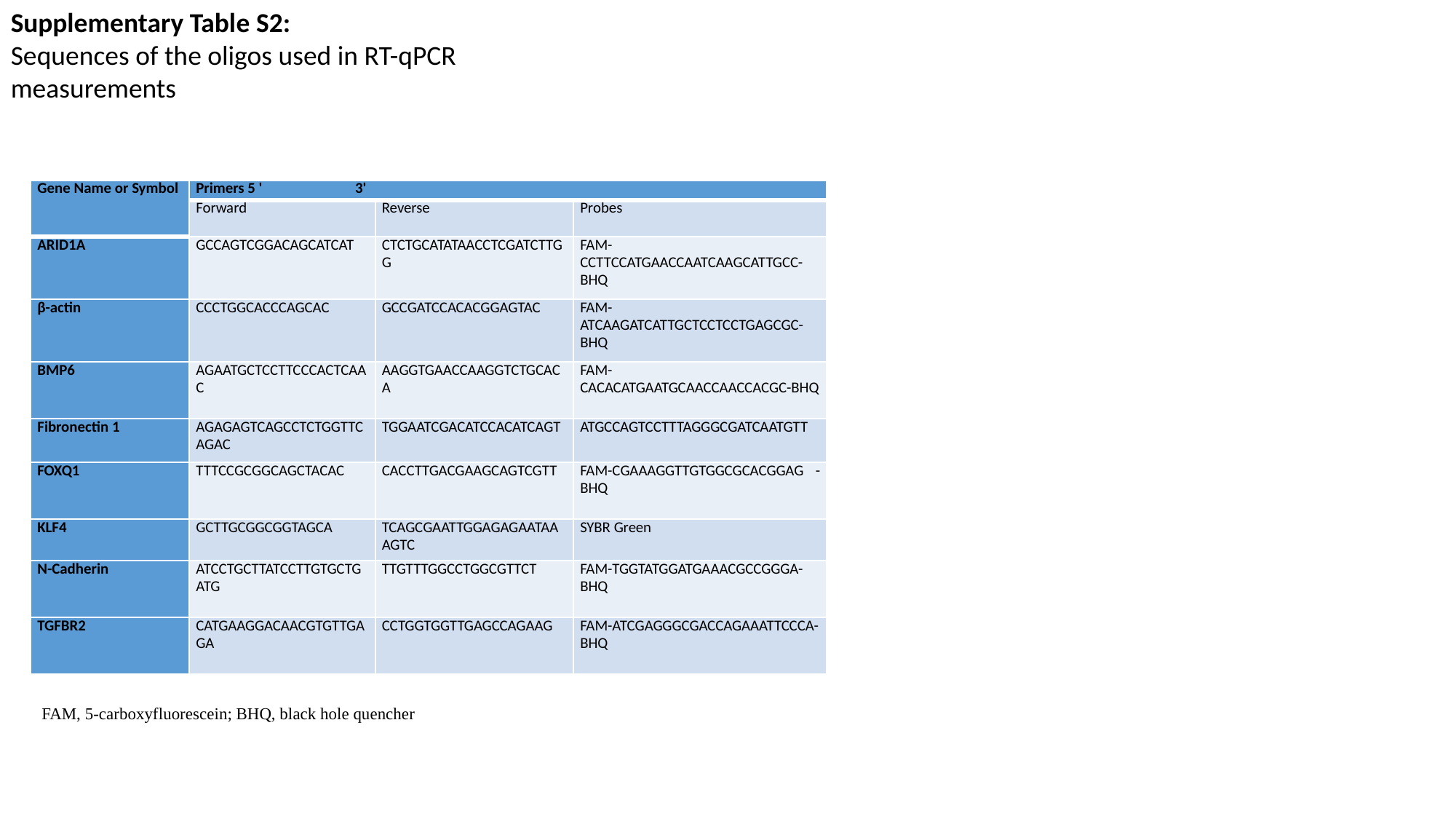

Supplementary Table S2:
Sequences of the oligos used in RT-qPCR measurements
| Gene Name or Symbol | Primers 5 ' 3' | | |
| --- | --- | --- | --- |
| | Forward | Reverse | Probes |
| ARID1A | GCCAGTCGGACAGCATCAT | CTCTGCATATAACCTCGATCTTGG | FAM-CCTTCCATGAACCAATCAAGCATTGCC-BHQ |
| β-actin | CCCTGGCACCCAGCAC | GCCGATCCACACGGAGTAC | FAM-ATCAAGATCATTGCTCCTCCTGAGCGC-BHQ |
| BMP6 | AGAATGCTCCTTCCCACTCAAC | AAGGTGAACCAAGGTCTGCACA | FAM-CACACATGAATGCAACCAACCACGC-BHQ |
| Fibronectin 1 | AGAGAGTCAGCCTCTGGTTCAGAC | TGGAATCGACATCCACATCAGT | ATGCCAGTCCTTTAGGGCGATCAATGTT |
| FOXQ1 | TTTCCGCGGCAGCTACAC | CACCTTGACGAAGCAGTCGTT | FAM-CGAAAGGTTGTGGCGCACGGAG -BHQ |
| KLF4 | GCTTGCGGCGGTAGCA | TCAGCGAATTGGAGAGAATAAAGTC | SYBR Green |
| N-Cadherin | ATCCTGCTTATCCTTGTGCTGATG | TTGTTTGGCCTGGCGTTCT | FAM-TGGTATGGATGAAACGCCGGGA-BHQ |
| TGFBR2 | CATGAAGGACAACGTGTTGAGA | CCTGGTGGTTGAGCCAGAAG | FAM-ATCGAGGGCGACCAGAAATTCCCA-BHQ |
FAM, 5-carboxyfluorescein; BHQ, black hole quencher

## Slide 5
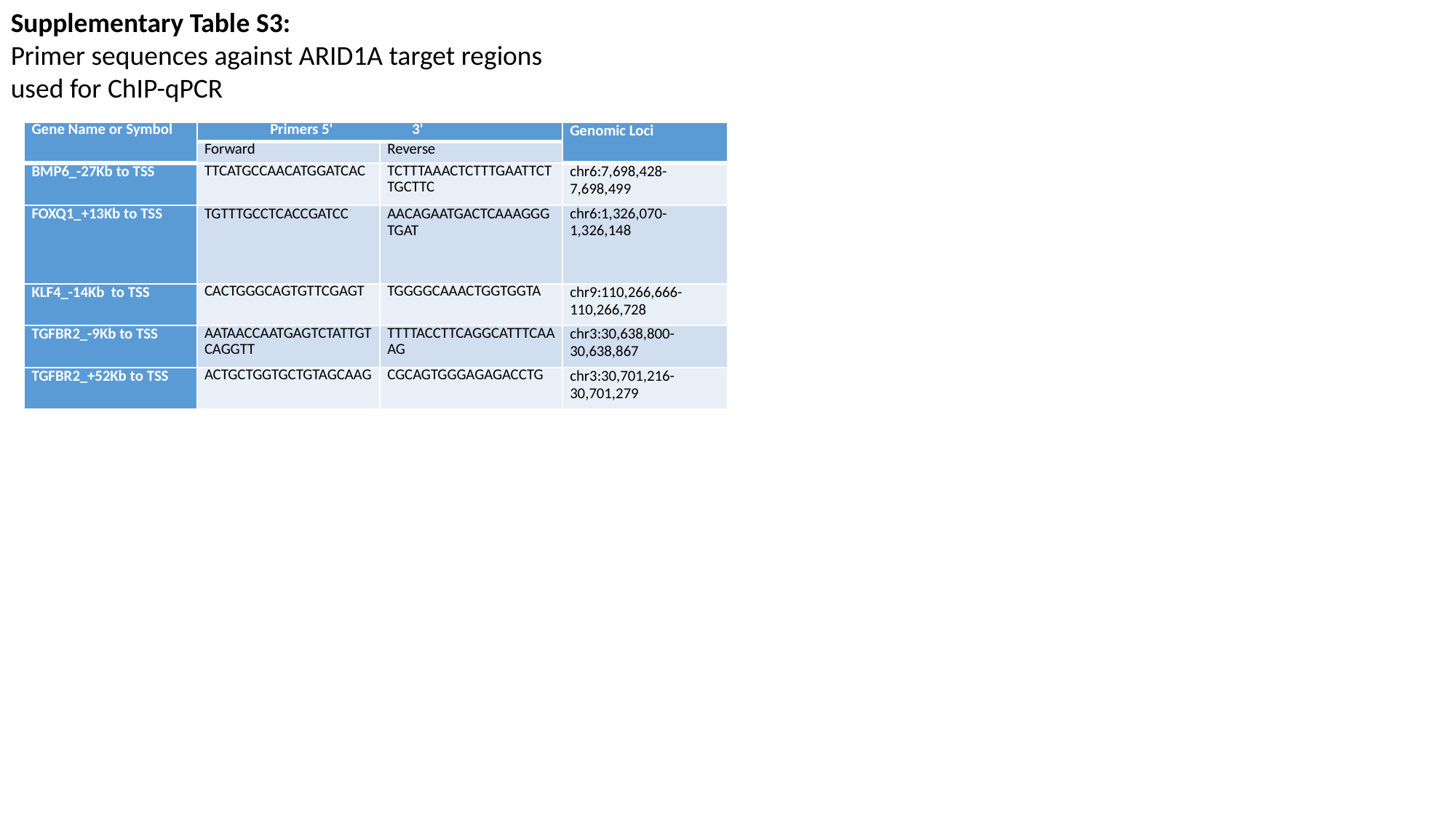

Supplementary Table S3:
Primer sequences against ARID1A target regions used for ChIP-qPCR
| Gene Name or Symbol | Primers 5' 3' | | Genomic Loci |
| --- | --- | --- | --- |
| | Forward | Reverse | |
| BMP6\_-27Kb to TSS | TTCATGCCAACATGGATCAC | TCTTTAAACTCTTTGAATTCTTGCTTC | chr6:7,698,428-7,698,499 |
| FOXQ1\_+13Kb to TSS | TGTTTGCCTCACCGATCC | AACAGAATGACTCAAAGGGTGAT | chr6:1,326,070-1,326,148 |
| KLF4\_-14Kb to TSS | CACTGGGCAGTGTTCGAGT | TGGGGCAAACTGGTGGTA | chr9:110,266,666-110,266,728 |
| TGFBR2\_-9Kb to TSS | AATAACCAATGAGTCTATTGTCAGGTT | TTTTACCTTCAGGCATTTCAAAG | chr3:30,638,800-30,638,867 |
| TGFBR2\_+52Kb to TSS | ACTGCTGGTGCTGTAGCAAG | CGCAGTGGGAGAGACCTG | chr3:30,701,216-30,701,279 |
